# Entropies of Negative Incomes, Pareto-Distributed Loss, and Financial Crises

**DOI:** 10.1371/journal.pone.0025053

**Published:** 2011-10-03

**Authors:** Jianbo Gao, Jing Hu, Xiang Mao, Mi Zhou, Brian Gurbaxani, Johnny Lin

**Affiliations:** 1 PMB Intelligence LLC, West Lafayette, Indiana, United States of America; 2 LNM, Institute of Mechanics, Chinese Academy of Sciences, Beijing, People's Republic of China; 3 Mechanical and Materials Engineering, Wright State University, Dayton, Ohio, United States of America; 4 Affymetrix, Inc., Santa Clara, California, United States of America; 5 Department of Electrical and Computer Engineering, University of Florida, Gainesville, Florida, United States of America; 6 Fredric G. Levin College of Law, University of Florida, Gainesville, Florida, United States of America; 7 School of Electrical and Computer Engineering, Georgia Institute of Technology, Atlanta, Georgia, United States of America; 8 Physics Department, North Park University, Chicago, Illinois, United States of America; University of Maribor, Slovenia

## Abstract

Health monitoring of world economy is an important issue, especially in a time of profound economic difficulty world-wide. The most important aspect of health monitoring is to accurately predict economic downturns. To gain insights into how economic crises develop, we present two metrics, positive and negative income entropy and distribution analysis, to analyze the collective “spatial” and temporal dynamics of companies in nine sectors of the world economy over a 19 year period from 1990–2008. These metrics provide accurate predictive skill with a very low false-positive rate in predicting downturns. The new metrics also provide evidence of phase transition-like behavior prior to the onset of recessions. Such a transition occurs when negative pretax incomes prior to or during economic recessions transition from a thin-tailed exponential distribution to the higher entropy Pareto distribution, and develop even heavier tails than those of the positive pretax incomes. These features propagate from the crisis initiating sector of the economy to other sectors.

## Introduction

Financial crises have been studied extensively through theoretical modeling [1], [2] and analysis of individual companies [3]–[6]. It is an open and important question whether the recent gigantic economic crisis can be analyzed using analogies from the physical world. In particular, it is important to determine whether it is a unique crisis entirely different from most other economic recessions or has quantifiable characteristics that are shared by other recessions and can be utilized to accurately predict it.

Phase transition-like behaviors have been reported in many systems outside of traditional thermodynamics, including two-phase behavior in the buying and selling in financial markets [7], and stock crashes [8]–[11]. Although thermodynamic analogies for economic systems have a storied, but contentious history [12], such a phenomenological description may provide clues to alternative metrics and methodologies of studying the collective behavior of an industry or economic systems in general. In the present study, we analyze two metrics related to such a paradigm, entropy and distribution changes, as applied to positive and negative incomes of U.S. companies in nine different sectors. Although our time series only contains quarterly data of 19 years, our analyses reveal “spatial” features of the data that can be otherwise hidden from more traditional analysis using fewer companies. In contrast to more traditional metrics [13], entropy and distribution changes are able to provide predictive skill for economic downturns with a negligible false-positive rate and capture the collective or aggregate dynamics of the development of downturns as weakness propagates from one sector to another. Both findings provide additional evidence to suggest that the phase transition paradigm may be a useful tool for analyzing the dynamics of economic systems.

## Materials and Methods

### 1. Data

We examine pretax quarterly incomes, from the beginning of 1990 to the end of 2008, of thousands of U.S. companies in 9 sectors: Financial, Consumer Goods, Consumer Services, Basic Materials, Health Care, Industrials, Oil/Gas, Tech-Telecommunications, and Utilities [14]. Prior to 1990, income data for many companies in these sectors are incomplete, and thus meaningful analysis is not feasible. In and after 2009, income data in some of these sectors have been profoundly altered due to strong governmental interference, and therefore objective analysis is not possible.

In the entropy and distribution metrics described below, we consider negative and positive incomes as separate clusters; when the number of companies in each category or cluster is sufficiently large, these metrics are able to characterize the stability of the clusters. Note, however, during certain episodes, such as the early part of the 1990 recession, corporate dislocations due to bankruptcy, mergers, etc., can result in underestimation of the number of companies with negative incomes, which produces a delayed identification of the onset of an economic downturn.

### 2. Shannon entropy

The stability or strength of an income cluster may be quantified by its entropy, given that the second law of thermodynamics can be equivalently restated as saying that the most stable configuration is the one with the highest entropy [15]. For the negative and positive income clusters considered here, the Shannon entropy is a pertinent measure, and for discrete probabilities is given by [16]:
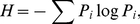
(1)where 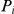 are the probabilities that the positive or negative incomes will fall within a prescribed bin 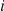 (where a bin is an interval of fixed length). We shall take 2 as the base of the logarithm so that the unit of the entropy is the bit. Note that when all the probabilities are equal, Shannon entropy attains its largest value; such a situation may be associated with the discretization of a uniform distribution.

### 3. Distributional analysis

In the distributional analysis, we shall focus on two types of distributions. One is the exponential distribution, whose complementary cumulative distribution function (CCDF) is given by
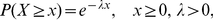
(2)The other is the Pareto distribution, whose CCDF is:
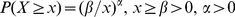
(3)where 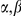 are parameters. Note that Eq. 3 is a special case of the heavy-tailed distribution, 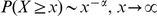. When 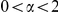, the distribution has infinite variance, and when 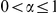, the mean is also infinite [17]. To fit real, finite data, one may use a truncated Pareto (or heavy-tailed) distribution.

## Results

### 1. Section-wide entropy analysis of income data

**Figure 1:**
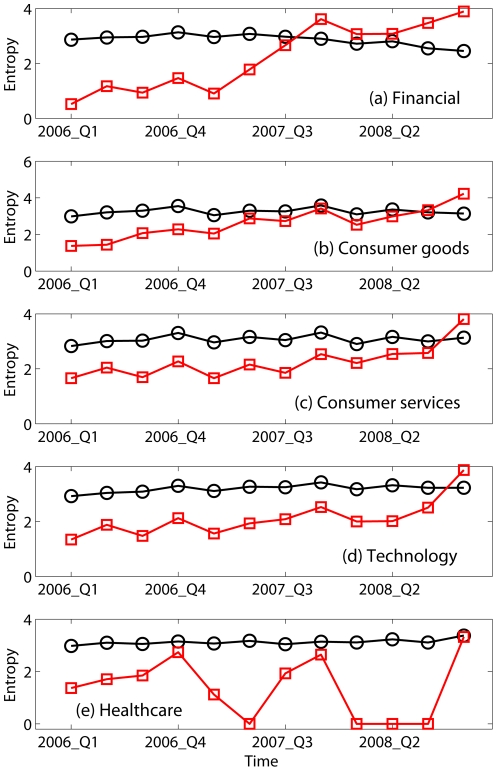
Entropies (in units of bits) for the distribution of positive (black circles) and negative (red squares) incomes from the first quarter of 2006 to the fourth quarter of 2008 for 5 sectors.

We analyze Shannon entropy 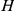 for the period preceeding and during the 2008 Financial Crisis, and find that this metric captures the dynamics of the onset of this crisis. Figure 1 shows 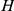 from the first quarter of 2006 to the fourth quarter of 2008, for positive (black circles) and negative (red squares) incomes in 5 sectors (Financial, Consumer Goods, Consumer Services, Technology, and Health Care). The discrete probabilities in Fig. 1 are computed using a bin size of $15 million. Tests using bin sizes of $5, $10, and $20 million shifted the curves vertically, but the difference between 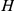 for positive and negative incomes is largely independent of the bin size. The entropy of the distribution of positive incomes is almost constant, for all five sectors examined here. In contrast, the entropy of the distribution of negative incomes varies considerably with time. For example, for the Financial sector, it is markedly smaller than the entropy of positive incomes until the third quarter of 2007, when the entropy rises sharply. By the third quarter of 2007, the difference between the two entropies is almost zero, suggesting that the cluster of financial companies with losses is almost as strong (i.e., this configuration is nearly as stable) as the cluster of profitable financial companies, and signals weakness in the sector. From the third quarter of 2007 on, the entropy of the distribution of negative incomes is noticeably larger than for positive incomes, indicating that the cluster of financial companies with negative incomes is well-established and stronger than the cluster of financial companies with positive incomes, consistent with the progression of the 2008 Financial Crisis.

The time series of entropy 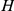 for other sectors in the period preceeding and during the 2008 Financial Crisis show behavior consistent with the leading role of the Financial sector in the crisis, and propagation of the crisis from that sector into other sectors of the economy. Consumer Services (Fig. 1(c)) and Technology (Fig. 1(d)), for instance, do not show noticeable weakness until after third quarter of 2008. The temporal variations of entropy for Consumer Goods (Fig. 1(b)) shows similar behavior, though in second quarter of 2007 the negative income entropy nearly surpasses positive income entropy, suggesting the beginning of sector weakness at that time.

**Figure 2:**
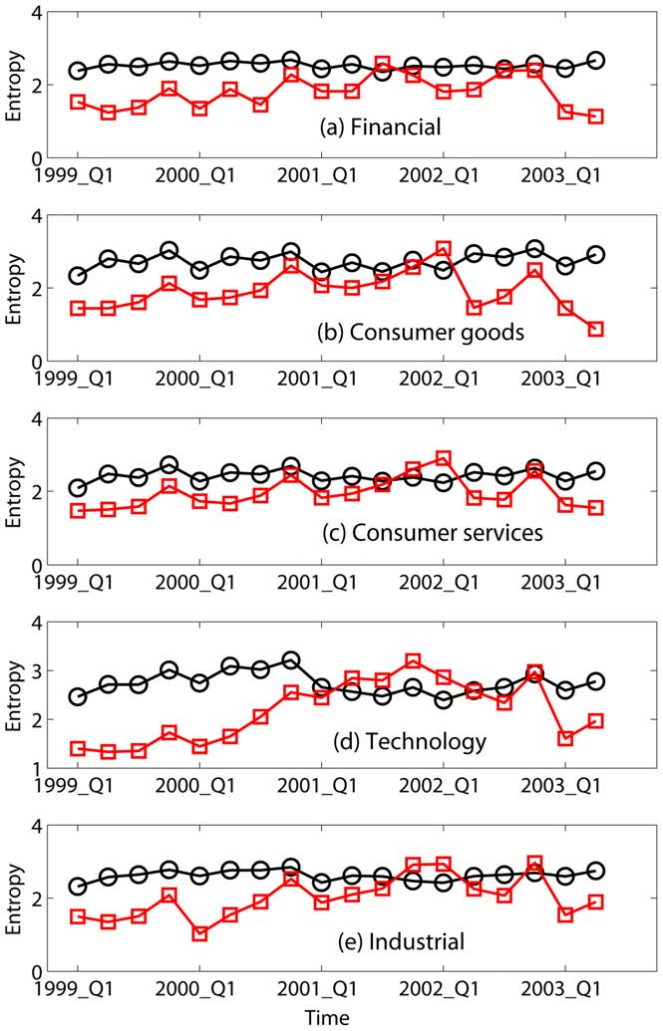
Entropies (in units of bits) for the distribution of positive (black circles) and negative (red squares) incomes from the first quarter of 1999 to the second quarter of 2003 for 5 sectors.

**Figure 3:**
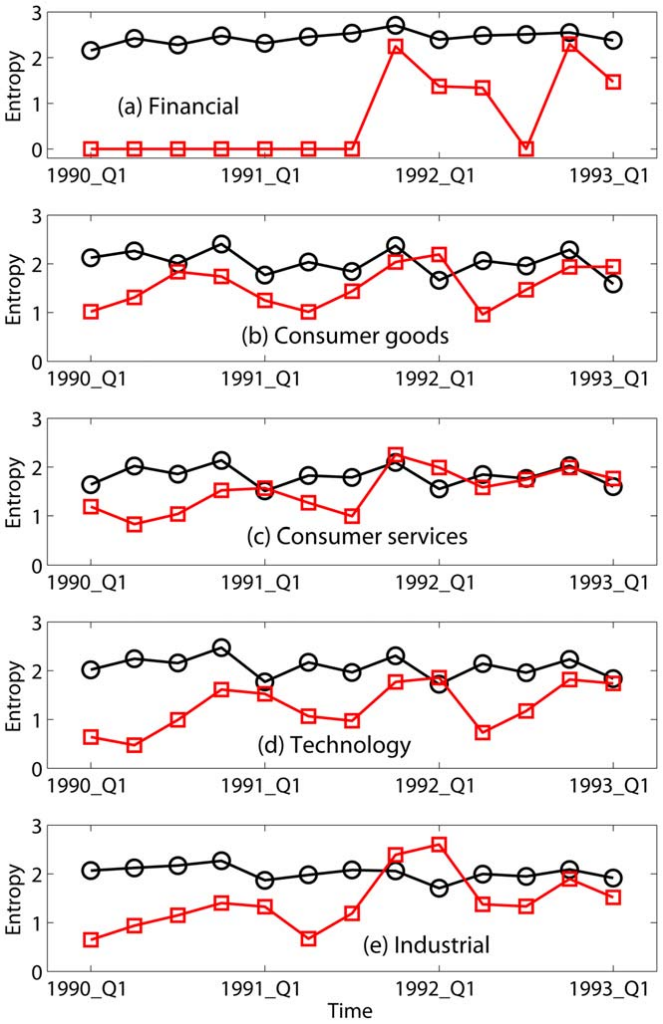
Entropies (in units of bits) for the distribution of positive (black circles) and negative (red squares) incomes from the first quarter of 1990 to the first quarter of 1993 for 5 sectors.

The propagation of weakness from one sector to another during the 2008 Financial Crisis, as revealed in positive and negative income entropy, is also seen during other periods of market decline, such as the 1999–2003 technology stock fueled decline (Fig. 2) and the early 1990 recession (Fig. 3). Therefore, at least over the 19 year record examined here, the behaviors seen in entropy are not unique features of the current crisis, but are rather common features shared by other periods of economic recession. Alternatively, we may say that the current crisis, though more serious than other recent recessions, from the entropy perspective differs in degree but not in kind.

**Figure 4:**
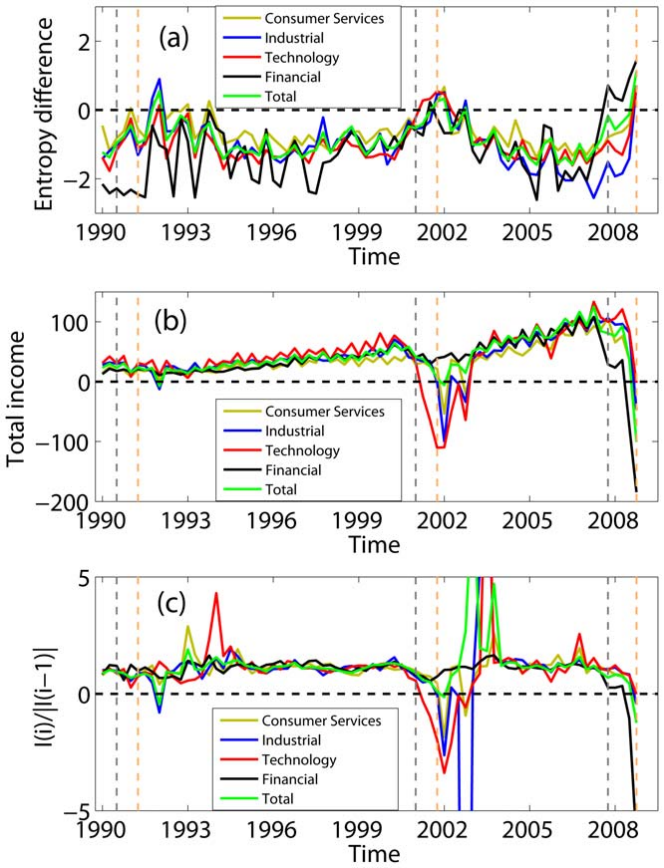
Identification of economic downturns by metrics such as difference between entropy of negative and positive incomes, total income, and total income ratio. (a) variation of difference between entropy of negative and positive incomes with time; for exact timing of recessions, see Figs. 1–3; (b) variation in time of total income (where the income of the 1st quarter of 1990 is taken as 1 unit); (c) variation in time of total income ratio 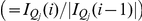, where 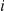 denotes year and 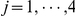 denotes quarter, so 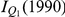 means 1st quarter income in 1990; this ratio crudely measures GDP contraction/expansion. The grey and orange vertical dashed lines indicate, respectively, the downturn onset times determined by NBER and the dates the NBER announced their onset identifications.

To quantitatively assess the skill of entropy in predicting economic downturns, we define the onset of a downturn as when the difference between the entropy of negative and positive incomes first becomes positive. By this criterion, using Figs. 1–3 and 4(a), we identify recession start times as the 4th quarter of 2007, 2nd quarter of 2001, and 1st quarter of 1991, respectively, with no false-positives (Fig. 4(a); note the three positive entropy differences indicated by Consumer Services in early 1990's all belonged to or indicated a continuation of the 1990 recession; none was a false-positive). This compares well with the National Bureau of Economic Research (NBER)'s business cycle contraction onset dating determinations during this period, which are 4th quarter of 2007, 1st quarter of 2001, and 3rd quarter of 1990 [18]; our identification of the downturn times matches the 2007 onset and is delayed by 1 and 2 quarters, respectively, for the 2001 and 1990 onsets. If we relax our criterion for onset of a downturn to be the first time when the entropy of negative income is 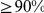 of positive income entropy, then our identified recession start times for the 2001 and 1990 downturns become 4th quarter of 2000, and 3rd quarter of 1990, with false-positives still zero. With this relaxed criterion, the entropy metric identifies downturn onset 0 quarter ahead, 1 quarter ahead, and 0 quarter ahead of the NBER onsets for the 2007, 2001, and 1990 downturns, respectively. The NBER business cycles determinations are, however, retrospectives. If we compare the dates of our entropy-based downturn onset identifications with the dates the NBER *announced* their onset identifications, our entropy-based identifications preceed the NBER announcements [18]; for the 2008 crisis, the entropy-based identification occurs 1 year earlier.

Additionally, because the NBER business cycle determinations are retrospective, they are not useful as comparators for evaluating the predictive skill of the entropy metric. To provide such a comparator, we define two “trivial” alternative metrics of recession onset: the time when total pretax income becomes negative, and the time when the ratio of incomes 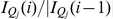 (where 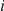 denotes year and 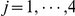 denotes quarter, so 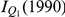 means 1st quarter income in 1990), is smaller than a certain threshold value. The first alternative metric provides a rudimentary snapshot of sector cash flow, while the second metric provides a crude measure of gross domestic product (GDP) contraction/expansion. In the case of the time series of total pretax income, recession times are identified with substantial longer delay than seen in entropy (Fig. 4(b)). (We note as an aside that the variation of the total pretax income seen around the 1990 recession suggests that the delayed identification of that recession by the entropy metric is likely due to underestimation of negative incomes, not the entropy metric itself.) The GDP contraction/expansion-like metric, either identifies similarly delayed recession start times, compared to those provided by total pretax income, or yields a substantial number of false-positives, depending on the threshold used to define downturns (Fig. 4(c)).

### 2. Mechanism of financial crises

**Figure 5:**
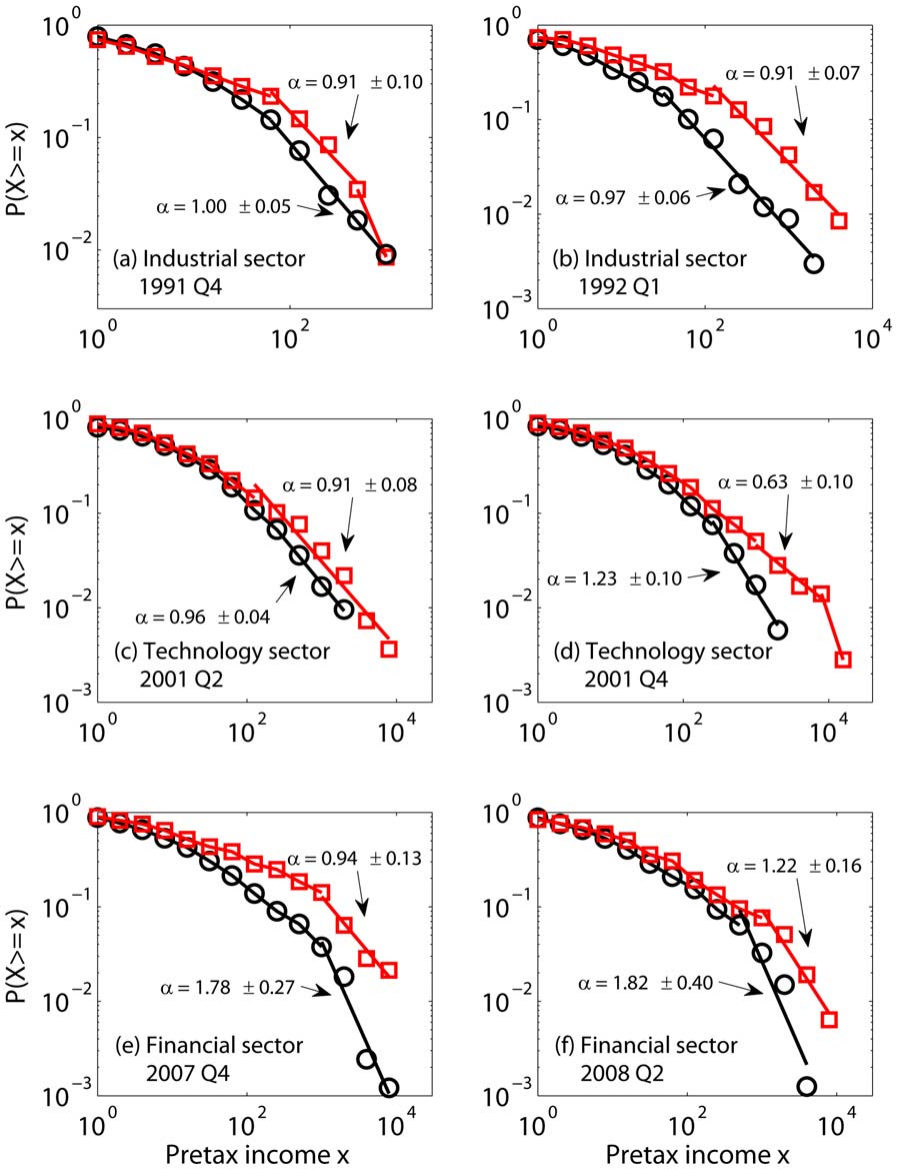
CCDF (log-log scale) for negative (red square) and positive (black circle) pretax incomes amongst U.S. companies. (a) industrial sector, fourth quarter of 1991, 116 and 327 negative and positive pretax incomes; (b) industrial sector, first quarter of 1992, 118 and 335 negative and positive pretax incomes; (c) technology sector, second quarter of 2001, 275 and 418 negative and positive pretax incomes; (d) technology sector, fourth quarter of 2001, 356 and 345 negative and positive pretax incomes; (e) financial sector, fourth quarter of 2007, 141 and 823 negative and positive pretax incomes; (f) financial sector, second quarter of 2008, 157 and 800 negative and positive pretax incomes.

The entropy variations shown in Figs. 1–3 and 4(a) suggest a phase transition propagation-like phenomenon where weakness begins in one sector and gradually propagates to others with different time delays. Entropy variations, however, do not yield much insight regarding the mechanism behind this transition-like behavior. Distribution analysis of positive and negative incomes provides tools for further analysis. Specifically, the equal-log-bin technique [17] is used to reliably estimate the CCDF from the sparse data in our time series. We find that like many other economic time series [19]–[22], positive income (denoted as 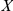) typically follows a Pareto distribution described by Eq. (3). The black circles in Figs. 5(a–f) are illustrative of positive income distributions in general, and the straight lines in the tail region of the log-log plots clearly indicate a heavy-tailed distribution. While the shape of the positive income distribution is largely independent of the economic health of the sector, the parameter 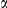 is not universal; instead, it correlates well with the health of the economy – it attains a larger value when a recession is more serious.

The shape of distribution of negative incomes, in contrast, strongly depends on the economic health of the sector. During healthy periods, negative incomes occur because of nonsystemic reasons (e.g., poor management) and are rare and small. When losses are very few, the loss distribution may not be well-defined; when losses become slightly more numerous, 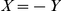 (where 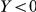 denotes negative incomes) may roughly follow an exponential distribution described by Eq. (2), or, with even more numerous losses, resemble a heavy-tailed distribution described by Eq. 3.

For the 2008 Financial Crisis, prior to the third quarter of 2007, the distribution of losses in any quarter is thinner than that of positive incomes in the same quarter (i.e., the tail of the loss distribution lies beneath the tail of the profit distribution). However, near the onset of and during a crisis, the distributions of negative incomes not only have also become Pareto, but have even heavier tails than those of positive incomes during the same period (Fig. 5(e,f)). Again, such behaviors is also seen in other recessions (Fig. 5(a–d)).

## Discussion

To summarize, the entropy variations shown in Figs. 1–2 suggest a phase transition propagation-like phenomenon where weakness begins in one sector and gradually propagates to others with different time delays. More importantly, it has been shown that the recent gigantic recession differs from earlier ones in degree but not in kind, and that entropy is an effective indicator of economic recessions. The favorable comparison of our entropy based recession predictions with those of NBER suggests that entropy can be en effective indicator for operational monitoring of economic health.

This transition from a largely non-Pareto distributed loss to a Pareto-distributed loss with an even heavier tail than for profits, in conjunction with a sharp contemporaneous increase in the entropy of the loss distribution, demonstrates a change akin to a phase transition in a physical system that undergoes large fluctuations with power-law behavior. (A similar phenomenon has been observed with earthquakes, where aftershocks are known to follow Omori's law [23], which is also a Pareto-like power law distribution under certain conditions.) The transition between these economic phases, as in thermodynamics, may depend on the dynamics of a network of interacting entities [7], [24], which in the economic case consists of companies connected via financial instruments, business relations, etc. The dynamics of the transition from profits to losses would thus be not only a function of external forcing (such as policy changes), but also the structure of the network (and of the negative income cluster in particular). The theory of evolutionary games may also shed light on the nature of the transitions [25].

While the above implications and the ultimate validity of the thermodynamic analogy is an open question, in the present work we have seen that the proposed entropy and distribution metrics appear to offer predictive skill and capture the dynamics of the propagation of weakness between economic sectors, and thus may offer an additional means of monitoring the health of the economy. Finally, the similarity between propagation of economic weakness and propagation in phase transitions suggests that the phase transition paradigm may be useful in understanding the dynamics of economic downturns.
